# Non-Alcoholic Fatty Liver Disease in Everyday Clinical Practice: From Diagnosis to Therapy

**DOI:** 10.3390/life15030363

**Published:** 2025-02-25

**Authors:** Marcin Kosmalski, Łukasz Mokros

**Affiliations:** 1 Department of Clinical Pharmacology, Medical University of Lodz, 90-153 Lodz, Poland; 2 Department of Child and Adolescent Psychiatry, Medical University of Lodz, 92-213 Lodz, Poland; lukasz.mokros@gmail.com

Non-alcoholic fatty liver disease (NAFLD) is currently one of the most common hepatic disorders observed in daily medical practice. With the incidence reaching over 30% in the population, the disease remains of interest to many healthcare sectors, posing a serious challenge to global public health. Due to its health-related, economic, and social consequences, it is of interest to the medical community (including nurses, dieticians, family doctors, internists, diabetologists, endocrinologists, hepatologists, bariatric surgeons, cardiologists, nephrologists, oncologists, psychiatrists, psychologists, gynecologists, pediatricians, and geneticists), as well as governmental and non-governmental institutions, both at the local and international level [1,2]. Despite the fact that the term NAFLD was first introduced in 1986, discussions concerning the definition, etiopathogenesis, diagnostics, and therapeutic possibilities of this disease entity are still going on, which is reflected in the increasing number of scientific publications [3,4]. The main theme of this discussion is undoubtedly the strong association between NAFLD and other metabolic disorders, such as obesity and carbohydrate and fat metabolism disorders [5]. The result of this correlation is a change in the acronym and diagnostic criteria for NAFLD and the introduction of the concept of metabolic dysfunction-associated steatotic liver disease (MASLD). It should be emphasized that the current new definition of NAFLD is gaining widespread support, as it is non-stigmatizing and its purpose is primarily to improve awareness and identification of patients with the disease [6].

Such a broad view of NAFLD has resulted in increasing amounts of new data on the mechanisms leading to excessive fat accumulation in the liver and the impact of this process on the functioning of other tissues and organs [7]. There is also still a great need to find reliable, non-invasive, and cost-effective methods enabling the diagnosis and assessment of the progression of NAFLD, including, above all, the diagnosis of non-alcoholic steatohepatitis (NASH) and, according to the new MASH terminology, metabolic dysfunction-associated steatotic liver disease, and the severity of the fibrosis process. The important role of diagnostic tools, including the markers of extrahepatic complications of NAFLD, should also be emphasized. It is suggested that they should bear the hallmarks of screening tools, i.e., tools characterized by fairly high sensitivity and specificity, and could be used on a large scale at the same time. To date, several non-invasive biomarkers (laboratory and imaging) based on single tests or scales with different degrees of reliability have been developed. In addition, the introduction of new NAFLD terminology has initiated a debate on the interchangeability of these terminologies and the need for more research into the variability of the results of these biomarkers [8,9]. Another rousing subject in this field is the lack of scientific evidence for the efficacy of pharmacological methods used to prevent and treat NAFLD, despite the available methods of non-pharmacological management in the case of diagnosis of that pathology [10]. These concerns are increasingly supported by strong evidence of a correlation between NAFLD and disorders such as obesity, type 2 diabetes mellitus (T2DM), cardiovascular disease (CVD), chronic kidney disease (CKD), obstructive sleep apnea, polycystic ovary syndrome (PCO), extrahepatic tumors, and osteoporosis, as well as personality factors [11,12].

Taking into account the growing interest in NAFLD and the complexities associated with the disease, the purpose of our Special Issue, entitled ”Non-alcoholic Fatty Liver in Everyday Clinical Practice: From Diagnosis to Therapy”, in the *Life* journal was to expand upon the knowledge in the broadest possible thematic areas of etiopathogenesis, diagnostics, complications, and possible therapeutic options to be used in patients with NAFLD. Our own observations indicate that this is necessary to improve the quality of medical care and to ensure the continuity of interdisciplinary care for patients diagnosed with this disease.

Eight thematically related articles were submitted to the editors, five of which received positive reviews and were accepted for publication. These papers were demonstrative in character, and analyzed each aspect of the scope of this Special Issue. They addressed new aspects of the development and progression of MASLD, including the involvement of adipokines [13] and coexistence with new disease entities such as sarcopenia [14], and highlighted the important elements of various forms of therapy, focusing primarily on comorbidities [15] and, in particular, antiplatelet therapy [16]. This Special Issue also included a paper concerning the need for cooperation between primary and specialist medical care facilities in order to provide coordinated management of a patient with MASLD and, thus, reduce complications, including hepatocellular carcinoma [17]. In addition, the authors of the articles highlighted the change in the definition of NAFLD, the increasing incidence of the disease, its progressive nature, and the available diagnostic methods [13,14,15,16,17].

So far, several theories have emerged regarding the correlations between excessive fatty infiltration of the liver and the presence of insulin resistance (IR), lipotoxicity, fat metabolism disorders, inflammatory process, and oxidative stress. It is worth noting that NAFLD is diagnosed histopathologically when more than 5% of hepatocytes are affected by steatosis. The process of fatty distension of hepatocytes seems to be very complex and goes beyond the organ itself. It is suggested that it is closely related to adipose tissue and the network of adipokines and cytokines involved in ensuring the homeostasis of lipid metabolism and the proper functioning of the liver [7]. Available data indicate that the activity of some of these substances, which are hormonal in nature, changes under the influence of fatty infiltration of the liver, and some of them only appear in the body following this infiltration [18]. Changes in the activity of adipokines in patients with NAFLD were addressed by Pezzino et al. in an article published in this Special Issue entitled ”Role of Perturbated Hemostasis in MASLD and Its Correlation with Adipokines”. The authors point to the important role of released adipokines in the regulation of energy expenditure, inflammation, and fibrosis in patients with MASLD, as well as their relationship with tissue- and organ-related complications of this disease and IR. They emphasized the important role of adipokines such as adiponectin, leptin, resistin, and ghrelin. In particular, they paid attention to the altered functioning of platelets, the coagulation system, and damage to the vascular endothelium, which may explain the organic complications of NAFLD/MASLD. The liver is a key organ playing an important role in the production and post-translational modification of coagulation factors, and its damage may contribute to the initiation of coagulation, which induces fibrogenesis, and vice versa. The clinical effect of this process is an increased risk of thrombotic and hemorrhagic events. This is due to the fact that increased levels of von Willebrand factor, fibrinogen, and factor VIII, and decreased antithrombin and protein C levels can be observed in the group of patients with MASLD. Elevated levels of plasminogen activator-1 inhibitor (PAI-1), combined with a decrease in tissue-activating factor antigen and tissue plasminogen activator antigen levels, which leads to a chronic state of hypofibrinolysis and, thus, an increased risk of disease mortality, are also of clinical significance. Taking into consideration cardiovascular complications in NAFLD patients, the authors of the study emphasize the proven presence of a negative correlation between platelet count and the occurrence of MASLD, as well as the association of this disease with the mean platelet volume (MPV) value. Additionally, they indicate the existence of impaired sinus endothelial function in the hepatic microcirculation due to the fact that the production of several substances and prothrombotic receptors, together with the recruitment of neutrophils and platelets, promotes the formation of an intrasinusoidal microthrombus, which leads to the destruction of parenchymal tissue and the progression of fibrosis. At the same time, the authors emphasize that endothelial disorders in MASLD are not limited to blood vessels in the liver but involve additional vascular networks, which are associated with increased susceptibility to cerebrovascular complications, including neurodegenerative complications in the brain and an increased likelihood of developing dementia [13]. It is worth noting that, as well as being potential non-invasive tools, the aforementioned markers of homeostasis are closely related to the inflammatory process, making it possible to establish the screening diagnosis of NAFLD, as well as assess disease progression and the effects of the instituted therapy [19,20].

Epidemiological data and known common pathomechanisms indicate a close relationship between NAFLD and other disease entities such as obesity, T2DM, dyslipidemia, CVD, CKD, cancer, and endocrine disorders, including hypothyroidism and osteoporosis [21]. The pathomechanism of NAFLD development, involving many other tissues and organs in addition to the liver, including adipose and muscle tissues, as well as insulin resistance (IR), inflammatory processes, genetic polymorphisms, and intestinal flora, is complex, and new disease entities closely related to fatty infiltration of the liver are still being sought after [22]. In our Special Issue, there is also an article by Ferens et al. which discusses the pathomechanical relationship between NAFLD and sarcopenia. The authors draw attention to sarcopenia as a proven and important factor predisposing individuals to the development and progression of NAFLD. Throughout this process, they have also identified its independence from other etiopathological factors such as IR, obesity, and inflammation—the three strongest predictors of NAFLD and its consequences. They also emphasize the bidirectional nature of the adverse consequences of the coincidence of NAFLD and sarcopenia, indicating an increased risk of symptomatic liver and kidney dysfunction, infections, carbohydrate metabolism disorders, osteoporosis, and, above all, mortality. Nevertheless, the authors emphasize the important role of IR (increased expression of forkhead box O family proteins, reduced synthesis of skeletal muscle proteins, increased catabolism, the process of autophagy occurring in skeletal muscle, gluconeogenesis promoting proteolysis, and reduced muscle mass increase IR), myosteatosis (sarcopenia, obesity, and infiltration of fat into muscle tissue result in a decrease in muscle size and increased muscle fat), and systemic inflammation, as well as disorders involving the secretion of myokines (irisin, myostatin, and adiponectin), vitamin D deficiency, low physical activity, and intestinal dysbiosis [14]. It should be emphasized that, for many reasons, this topic is very important from the clinical point of view. It is based on the common problem of malnutrition in patients with sarcopenia and liver cirrhosis due to NAFLD and examines the deterioration of their quality of life, as well as unfavorable prognoses [23,24,25].

NAFLD is a significant public health problem due to its increasing prevalence, the changing concepts used to define this disease, its complex etiopathogenesis, its frequent coexistence with other disease entities, the current lack of effective pharmacotherapy methods, and, above all, its complex diagnostic methodology. All of these factors necessitate close cooperation of the individual healthcare sectors in order to prevent complications of NAFLD, including hepatocellular carcinoma (HCC). An analysis of this cooperation was presented in this Special Issue in an article by Nagai et al., entitled “Frontiers of Collaboration between Primary Care and Specialists in the Management of Metabolic Dysfunction-Associated Steatotic Liver Disease: A Review”. The authors of the paper emphasized that primary care physicians must always be careful not to overlook cases of MASLD with advanced fibrosis. For this purpose, they implemented a simple and non-invasive testing method (NIT), including biochemical blood tests based on direct markers (type IV collagen 7 s domain, ELF test, hyaluronic acid, Mac2-binding protein glucosylation isomer (M2BPGi), and type III procollagen peptide (PIIIP)) and indirect markers (fibrosis-4 (FIB-4) index, NAFLD fibrosis score (NFS), and platelets). It should be emphasized that due to insufficient diagnostic efficiency, no direct markers have been approved. The FIB-4 index, based on AST, ALT, PLT, and patient age, has gained prominence as the first screening method used by primary care physicians to detect advanced fibrosis in patients with MASLD [17], which is in line with the current guidelines from global societies dealing with this disease [10]. At the same time, the need to refer patients to specialist care (hepatological and gastrological) in the case of FIB-4 values ≥ 1.3 is emphasized in order to extend the diagnosis to include elastography (vibration-controlled transient elastography (VCTE) or ultrasound elastography) or liver biopsy, and, in selected clinical situations, magnetic resonance elastography (MRE). Patients with MASLD and without advanced liver fibrosis should be placed under the regular care of a family doctor with the need to reassess FIB-4 every 2–3 years in patients without metabolic risk factors and every 1–2 years in patients with metabolic risk factors. Collaboration between GPs and hepatologists is necessary at every stage of the disease process, from control examination and secondary screening to treatment and follow-up. At the same time, they emphasize that the differences in goals, education, and cultures, as well as a lack of understanding of other professions, may be the reasons for the difficulties in cooperation between primary care physicians and hepatologists [17].

The therapeutic options available in the case of NAFLD diagnosis are aimed at health-promoting activities, including diet and physical exercise aimed at weight reduction. At the moment, there is a shortage of registered drugs that directly target NAFLD. Additionally, much attention is paid to comorbidities, especially obesity, dyslipidemia, and type 2 diabetes, which are the basis for the diagnosis of MASLD [26]. In the paper by Elshaer et al., submitted to our Special Issue, entitled “Exploring Varied Treatment Strategies for Metabolic Dysfunction-Associated Steatotic Liver Disease (MASLD)”, the authors present, in detail, the therapeutic options which may be used in patients with MASLD. In particular, they emphasize that such treatment should focus on effectively addressing the underlying risk factors for development of the disease and preventing further progression of the disease. In addition, they highlight that early diagnosis and treatment have the potential to reduce the risk of complications and associated morbidity and mortality. In addition to diet and exercise, which have a documented significant beneficial effect on the clinical and histopathological picture of fatty liver, the authors discussed the effectiveness of available diets. They highlight the effectiveness of the Mediterranean diet, the DASH diet (Dietary Approaches to Stop Hypertension), and a calorie-reduced diet. Various other dietary approaches such as vegan, low-carbohydrate, and low-fat diets, early feeding time restrictions, and intermittent fasting have also been investigated for the treatment of MASLD. However, these trials were limited by the lack of liver biopsies and the heterogeneity of the study cohorts. In conclusion, dietary recommendations should encourage the implementation of a macronutrient-dense diet with limited carbohydrates and saturated fats, such as the Mediterranean diet. A diet plan should focus on achieving weight loss through a balanced long-term diet plan. Such a holistic approach ensures that the dietary intervention is tailored to individual preferences with lasting health benefits. In addition, they emphasize the role of alcohol consumption in the progression of MASLD and the beneficial effects of coffee in this group of patients, as well as its protective properties against the development and progression of the disease. It is noteworthy that the consumption of regular filter coffee has a more protective effect on the liver and reduces fibrosis than the consumption of espresso, which is probably related to the light roasting of regular coffee and, thus, the retention of compounds such as chlorogenic acids. The authors of the paper also discuss the usefulness of vitamin E, pioglitazone, incretinomimetics, flozins, aspirin, pemafibrate, resmetirone, statins, metformin, phentermine, naltrexone/bupropion, orlistat, and phentermine/topiramate, as well as bariatric surgery, in patients with MASLD [14].

The presence of MASLD is a risk factor for the development of complications, both from the liver itself and arising from systemic issues, including cardiovascular ones. In addition, the correlation with the prothrombotic state is discussed. Therefore, it seems justified to discuss the indications for the use of antiplatelet therapy in this group of patients. Such a discussion was undertaken by Boccatonda et al. in their article submitted to our Special Issue. They emphasize the presence of increased aggregability and activation of PLT, associated, among others, with leptin, circulating platelet-derived microvesicles (PMVs), and liver thromboxane (TX) A2 levels, as well as hypertriglyceridemia and insulin resistance. They describe in detail the potential role of PLT in the pathogenesis of liver disease and inflammation highlighted by immunohistochemical staining on liver biopsy specimens, which demonstrated the presence of great amounts of platelet and neutrophil extracellular traps (NETs) in the liver, with a relation to NAFLD activity score. It is also important that this morphotic element, along with the values of platelet distribution width (PDW) and mean platelet volume (MPV), can be used to assess the risk of development of liver fibrosis. Following these suggestions, the authors of the study present data on the possibilities of antiplatelet therapy as a method to alleviate the development of fibrosis in NAFLD (as an antifibrotic agent). One of the complications of this process is the development of hepatocellular carcinoma. Describing the possible mechanisms responsible for this effect, the authors also point to the controversy between ASA and NSAID in this regard. In addition, they emphasize that in the literature, there are no specific and extensive studies on the administration of antiplatelet therapy in people with fatty liver disease or in patients covered by the new definition of MASLD. Most studies involve the administration of antiplatelet therapy to patients with multiple cardiometabolic risk factors, and, in particular, with diabetes. Aspirin monotherapy is commonly recommended for secondary prevention of atherosclerotic cardiovascular events in both diabetic and non-diabetic patients, and antiplatelet therapy should be considered in individuals with a high cardiovascular risk profile. As far as the use in patients with MASLD is concerned, they emphasize that, at present, the institution of antiplatelet therapy in patients with MASLD is based mainly (and often solely) on the evaluation of cardiometabolic comorbidities, with no reference to the degree of hepatic alteration; this is due to the lack of strong evidence and adequate studies [16].

## Conclusions

This Special Issue presents the current state of knowledge in the field of NAFLD/MASLD and, above all, indicates the practical aspects of care for this group of patients with regard to comorbidities and the risks associated with the diagnosis of this disease, as well as its diagnostic and therapeutic options. It further emphasizes the complexity of NAFLD/MAFLD, which is associated with the need for further research in every direction related to this disease. In particular, the prevalence of cardiovascular risk in this group of patients should be emphasized, hence the need for this Special Issue in the *Life* journal, entitled “Metabolic Health: The Interplay between NAFLD, MAFLD, MASLD and Cardiovascular Disease”.
